# Draft genome sequence of *Acinetobacter junii* strain ABJ_A23_1 isolated from soil in Gwagwalada, Nigeria

**DOI:** 10.1128/mra.00160-25

**Published:** 2025-05-20

**Authors:** Ahmed Olowo-okere, E. Skiebe, G. Wilharm

**Affiliations:** 1 Department of Pharmaceutical Microbiology and Biotechnology, Faculty of Pharmaceutical Sciences, University of Abuja 99399, Abuja, Federal Capital Territory, Nigeria; 2 Project group P2, Robert Koch Institute, Wernigerode, Germany; DOE Joint Genome Institute, Berkeley, California, USA

**Keywords:** *Acinetobacter junii*, soil, draft genome, Nigeria

## Abstract

We report the draft genome sequence of *Acinetobacter junii* strain ABJ_A23_1 isolated from soil in Gwagwalada, Nigeria. The genome has a total size of 3,200,723 bp, 38.48% GC content, and 3,075 genes, including 2,975 protein-coding sequences, 73 RNA genes, and an integrative conjugative element.

## ANNOUNCEMENT

The genus *Acinetobacter* comprises gram-negative, non-fermentative bacteria widely distributed in various environments (1, 2). *Acinetobacter junii* is a species within this genus that has been less studied compared to its clinically significant relatives in the *Acinetobacter calcoaceticus-Acinetobacter baumannii* complex (3). However, *A. junii* has emerged as an ecologically diverse, One Health pathogen that has been implicated in clinical infections (4, 5). In this study, we report the draft genome sequence of *A. junii* strain ABJ_A23_1 isolated from soil collected in Gwagwalada, Nigeria.

*Acinetobacter junii* strain ABJ_A23_1 was isolated from a stream sediment sample collected in Passo, Gwagwalada, Nigeria (8°58′51″N, 7°03′04″E; altitude, 177.64 m) in August 2024. The sample was pre-enriched in mineral salt medium supplemented with 0.2% sodium acetate for 5 h at 37°C and subsequently plated on CHROMagar Acinetobacter and incubated overnight at 37°C (6). Bright red colonies with morphology typical of *Acinetobacter* species were selected for further characterization. Genomic DNA was extracted from one of the colonies using the MasterPure DNA Purification Kit (Epicentre) and quantified using the Invitrogen Qubit Assay. Sequencing of the genome on the Illumina MiSeq platform generated 13,398,908 paired-end reads (2 × 150 bp). The genome sequence was *de novo* assembled using the SPAdes version 4.0 algorithm in shovill version 1.1.0 (7, 8). The completeness, contamination, and genomic metrics were assessed using checkm2 version 1.0.2 and QUAST version 5.2.0 (9, 10). The genome functional annotation was performed using the NCBI Prokaryotic Genome Annotation Pipeline (11). The taxonomic position of the strain was confirmed through average nucleotide identity (ANI) analysis against the type strain of *A. junii* (12). Subsequently, antibiotic resistance genes were identified using ResFinder and the Comprehensive Antibiotic Resistance Database (CARD) Resistance Gene Identifier (13, 14). A web-based tool, ICEfinder version 3.0, was used to detect integrative and conjugative elements (ICEs) in the assembled genome (15).

The assembly resulted in 126 contigs with an *N*_50_ of 62,941 bp. The completeness of the genome of *A. junii* ABJ_A23_1 was estimated at 100%, with a contamination level of 0.09%. The draft genome has a total size of 3,200,723 bp, with a GC content of 38.48%. The average gene length is 311 bp. The NCBI PGAP annotation of the genome of *A. junii* ABJ_A23_1 identified a total of 3,075 genes, including 2,975 protein-coding genes and 73 RNA genes (64 tRNAs and 4 non-coding RNAs). A CRISPR array and an additional 27 pseudogenes were predicted (Table 1). ANI analysis revealed a 97.42% identity between the genome of strain ABJ_A23_1 (ASM4490305v1) and *A. junii* NCTC10307^T^ (46338_C01). This ANI value exceeds the species delineation threshold of 95%, confirming *A. junii* ABJ_A23_1 as a member of the *A. junii* species. While antibiotic resistance gene (ARG) prediction using ResFinder did not detect any ARG, including *bla*_OXA_ genes, which are intrinsic to the genomes of many *Acinetobacter* species, CARD Resistance Gene Identifier analysis predicted the presence of a small multidrug resistance antibiotic efflux pump (*qacG*) conferring resistance to disinfecting agents and antiseptics. ICEberg version 3.0 analysis detected a 143,064 bp putative ICE with a type IV secretion system spanning between positions 2,229,798 and 2,372,861 bp.

**TABLE 1:** Assembly and genome annotation metrics for *A. junii* ABJ_A23_1

Metric	Value
Completeness	100
Contamination	0.09
Genome size (bp)	3,200,723
GC content (%)	38.48
Number of contigs	126
Contig *N*_50_ (bp)	62,941
Contig *N*_90_ (bp)	21,165
*L*_50_	13
Total genes	3,075
CDSs (total)	3,002
Total coding genes	2,975
Total RNA	73
rRNA	5
tRNA	64
Repeat regions	1

## Data Availability

Genome sequence reads have been deposited in the Sequence Read Archive under the BioProject accession PRJNA1244528 and SRR32928652. The Whole-Genome Shotgun project has also been deposited in the DDBJ/ENA/GenBank under the accession JBJDIY000000000. The version described in this paper is JBJDIY000000000.

## References

[1] Wilharm G, Skiebe E, Łopińska A, Higgins PG, Weber K, Schaudinn C, Neugebauer C, Görlitz K, Meimers G, Rizova Y, et al.. 2024. On the ecology of Acinetobacter baumannii – jet stream rider and opportunist by nature. bioRxiv. doi:10.1101/2024.01.15.572815

[2] Pulami D, Schauss T, Eisenberg T, Blom J, Schwengers O, Bender JK, Wilharm G, Kämpfer P, Glaeser SP. 2021. Acinetobacter stercoris sp. nov. isolated from output source of a mesophilic german biogas plant with anaerobic operating conditions. Antonie Van Leeuwenhoek 114:235–251. doi:10.1007/s10482-021-01517-7 33591460 PMC7902594

[3] Attili A-R, Nocera FP, Sisto M, Linardi M, Gigli F, Ngwa VN, Fiorito F, Cerracchio C, Meligrana MCT, Bonacucina E, Cuteri V, De Martino L. 2024. Evidence and antibiotic resistance profiles of clinical Acinetobacter calcoaceticus-Acinetobacter baumannii (ACB) and non-ACB complex members in companion animals: a 2020–2022 retrospective study. Comp Immunol Microbiol Infect Dis 109:102185. doi:10.1016/j.cimid.2024.102185 38663213

[4] Boudjella-Senhadji I, Bour M, Potron A, Lorme F, Belal-Khedim L, Razafimahefa H, Lecointe D. 2024. Cross-transmission of Acinetobacter junii carrying bla_OXA-58_ in a neonatal ICU. J Antimicrob Chemother 79:1910–1913. doi:10.1093/jac/dkae180 38958235

[5] Aguilar-Vera A, Bello-López E, Pantoja-Nuñez GI, Rodríguez-López GM, Morales-Erasto V, Castillo-Ramírez S. 2024. Acinetobacter junii: an emerging One Health pathogen. mSphere 9:e0016224. doi:10.1128/msphere.00162-24 38606973 PMC11237400

[6] Yusuf I, Skiebe E, Wilharm G. 2023. Evaluation of CHROMagar Acinetobacter and MacConkey media for the recovery of Acinetobacter baumannii from soil samples. Lett Appl Microbiol 76:ovac051. doi:10.1093/lambio/ovac051 36695425

[7] Bankevich A, Nurk S, Antipov D, Gurevich AA, Dvorkin M, Kulikov AS, Lesin VM, Nikolenko SI, Pham S, Prjibelski AD, Pyshkin AV, Sirotkin AV, Vyahhi N, Tesler G, Alekseyev MA, Pevzner PA. 2012. SPAdes: a new genome assembly algorithm and its applications to single-cell sequencing. J Comput Biol 19:455–477. doi:10.1089/cmb.2012.0021 22506599 PMC3342519

[8] Seemann T. 2020. Shovill: assemble bacterial isolate genomes from Illumina paired-end reads. Github. https://github.com/tseemann/shovill.

[9] Gurevich A, Saveliev V, Vyahhi N, Tesler G. 2013. QUAST: quality assessment tool for genome assemblies. Bioinformatics 29:1072–1075. doi:10.1093/bioinformatics/btt086 23422339 PMC3624806

[10] Chklovski A, Parks DH, Woodcroft BJ, Tyson GW. 2023. CheckM2: a rapid, scalable and accurate tool for assessing microbial genome quality using machine learning. Nat Methods 20:1203–1212. doi:10.1038/s41592-023-01940-w 37500759

[11] Tatusova T, DiCuccio M, Badretdin A, Chetvernin V, Nawrocki EP, Zaslavsky L, Lomsadze A, Pruitt KD, Borodovsky M, Ostell J. 2016. NCBI prokaryotic genome annotation pipeline. Nucleic Acids Res 44:6614–6624. doi:10.1093/nar/gkw569 27342282 PMC5001611

[12] Jain C, Rodriguez-R LM, Phillippy AM, Konstantinidis KT, Aluru S. 2018. High throughput ANI analysis of 90K prokaryotic genomes reveals clear species boundaries. Nat Commun 9:5114. doi:10.1038/s41467-018-07641-9 30504855 PMC6269478

[13] Alcock BP, Huynh W, Chalil R, Smith KW, Raphenya AR, Wlodarski MA, Edalatmand A, Petkau A, Syed SA, Tsang KK, et al.. 2023. CARD 2023: expanded curation, support for machine learning, and resistome prediction at the Comprehensive Antibiotic Resistance Database. Nucleic Acids Res 51:D690–D699. doi:10.1093/nar/gkac920 36263822 PMC9825576

[14] Florensa AF, Kaas RS, Clausen PTLC, Aytan-Aktug D, Aarestrup FM. 2022. ResFinder – an open online resource for identification of antimicrobial resistance genes in next-generation sequencing data and prediction of phenotypes from genotypes. Microb Genom 8:000748. doi:10.1099/mgen.0.000748 35072601 PMC8914360

[15] Wang M, Liu G, Liu M, Tai C, Deng Z, Song J, Ou H-Y. 2024. ICEberg 3.0: functional categorization and analysis of the integrative and conjugative elements in bacteria. Nucleic Acids Res 52:D732–D737. doi:10.1093/nar/gkad935 37870467 PMC10767825

